# Travel distance, frequency of return, and the spread of disease

**DOI:** 10.1038/s41598-023-38840-0

**Published:** 2023-08-28

**Authors:** Cate Heine, Kevin P. O’Keeffe, Paolo Santi, Li Yan, Carlo Ratti

**Affiliations:** 1 Senseable City Lab, Massachusetts Institute of Technology, Cambridge, MA 02139 USA; 2 Istituto di Informatica e Telematica del CNR, Pisa, Italy

**Keywords:** Ecological modelling, Ecological networks

## Abstract

Human mobility is a key driver of infectious disease spread. Recent literature has uncovered a clear pattern underlying the complexity of human mobility in cities: $r \cdot f$, the product of distance traveled *r* and frequency of return *f* per user to a given location, is invariant across space. This paper asks whether the invariant $r\cdot f$ also serves as a driver for epidemic spread, so that the risk associated with human movement can be modeled by a unifying variable $r\cdot f$. We use two large-scale datasets of individual human mobility to show that there is in fact a simple relation between *r* and *f* and both speed and spatial dispersion of disease spread. This discovery could assist in modeling spread of disease and inform travel policies in future epidemics—based not only on travel distance *r* but also on frequency of return *f*.

## Introduction

The global impact of the COVID-19 pandemic on both human health and socioeconomic activity has brought to light the importance of a nuanced understanding of the way that epidemics spread in cities. Urban spread of disease is inherently tied to human mobility: as we move through and between cities, we serve as vectors that allow disease to spread to new individuals and communities. The nature of the relationship between mobility and spread of disease has been extensively studied; it is widely understood that human travel is a driving force behind disease spread^1–5^. For this reason, many public policy interventions implemented worldwide to contain the spread of COVID-19 focused on limiting mobility, restricting travel between regions and countries and encouraging individuals to avoid unnecessary travel^6–10^.

A large body of dynamic transmission modelling literature has incorporated spatial structure and mobility patterns into models of disease spread—for example, modelling the spread of disease across random spatial networks^11^, incorporating human movement patterns into metapopulation models of disease^12^, or assessing the impact of different types of movement in spatial-transmission models^13^. Accurate understandings of human mobility are critical to this literature, as different models of mobility can produce significantly different expected disease dynamics^14^. As we continue to learn more about how humans move, it is important to apply new discoveries about human mobility to the study of disease spread, deepening our understanding of urban epidemics for use in future public health crises.

Recent research uncovered a clear pattern in urban mobility: the total distance that the average visitor to a given location travels to reach it is constant across a city, unrelated to the location’s overall attractiveness ^15^. This value, equivalent to the distance of a location to an individual’s home multiplied by the number of times that they visit it over the course of some time period, can be thought of as an “exploration velocity”: it is the effective distance individuals travel towards any given location per unit of time. It is a simple but critical parameter to understanding human movement.

Given the simplicity of this parameter of human mobility and its apparent consistency across urban contexts, its relationship to disease spread is a natural next question. What if speed of disease spread depends not just on radius of travel *r*, but also on the product $v:= r\cdot f$? If *v* has additional impact on disease spread, beyond average travel radius *r*, mobility models that do not incorporate this dependency may fail to accurately model disease spread through urban systems. This paper investigates the relationship between exploration velocity $v:= r\cdot f$ and spread of disease, opening the door to future research on the ways in which that relationship can be utilized to model and contain epidemics.

## Results

### Simulating reduction of exploration velocity on real data

In order to understand how $v:= r\cdot f$ interacts with speed of disease spread, we start with large-scale datasets of individual human movements in New York City and Dakar, Senegal, which we call $\mathscr{M}_{\text {real}}$. The datasets each consist of a set of trips for *N* individuals over different time periods *T*, where each trip indicates a given individual moving between two locations. We then use agent based susceptible-infected (SI), susceptible-infected-recovered (SIR), and susceptible-exposed-infected-recovered (SEIR) models calibrated with transmission parameter estimates for COVID-19^16^ to simulate disease spread as agents follow the trajectories in our datasets. Aside from their unique trajectories, each individual is assumed identical. In each simulation, we vary two parameters: the maximum travel distance *r*, measured relative to each agent’s home location (see Methods for details), and the travel frequency *f*, the maximum number of times each location was visited. By varying these two parameters together, we are able to manually adjust exploration velocity *v* in the system. In practice, this means discarding any trip of length greater than *r* and all but *f* randomly selected trips to a given location from our datasets. Varying *r* and *f* separately allows us to isolate their individual effects on simulation outcomes—see Figure S1 in the Supplementary Material for results of simulations in which we vary the product $r\cdot f$ itself. Simulations are run over the entire length of the datasets (28 days for the New York City dataset; 14 days for the Dakar dataset) with 10,000 agents. The details of the spatial partitionings used as well as other simulation details are given in the Methods.

**Figure 1:**
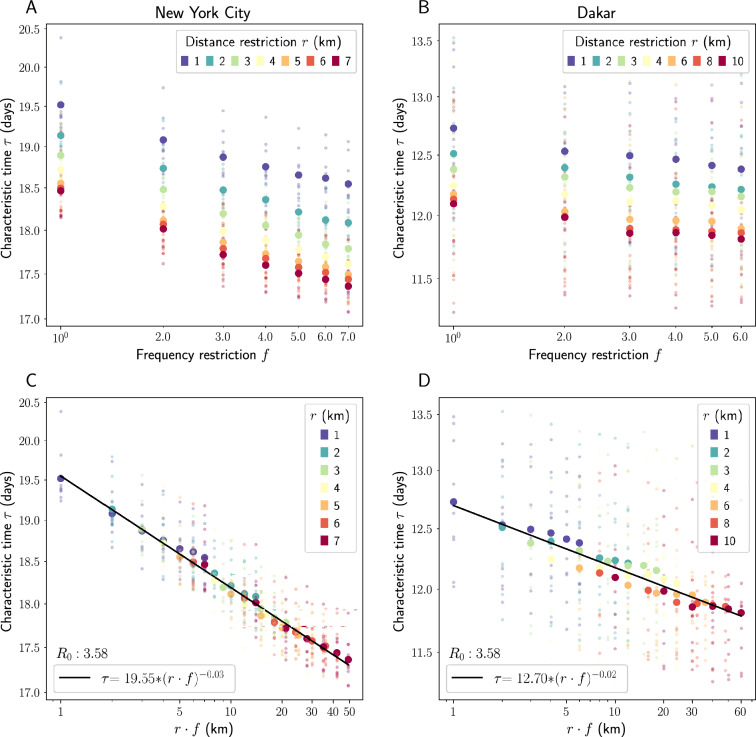
Characteristic times for SEIR model. Top row: characteristic time plotted against maximum travel frequency *f* for different values of maximum allowed travel distance *r*. Bottom row: characteristic time in days plotted against exploration velocity $r\cdot f$. All plots have logarithmic scales on both the x and y axes. Each smaller data point represents the result of one of 10 total simulations, with the larger data points representing their average. For simulation details see Methods. $R^2$ values for best-fit lines are .990 (NYC) and .917 (Dakar). Best-fit line parameters are $a = -0.03, b = 19.55$ (NYC) and $a = -0.02, b = 12.70$ (Dakar).

Figure 1A and B plot *characteristic time* $\tau$, the time that the disease process takes to reach $1/e \approx 36\%$ of all susceptible individuals in our SEIR models, against our frequency restriction, the number of times agents are allowed to return to the same place. The trends are intuitive and not surprising. For a given *f*, $\tau$ decreases monotonically with *r*: the further people are allowed travel, the faster the epidemic spreads. Similarly, for a given *r*, $\tau$ decreases monotonically with *f*: more frequent return trips lead to faster-spreading epidemics. What is surprising, however, is that the $\tau (r)$ curves for each value of *f* have similar shape. This echoes a previous finding^15^ and hints that $\tau , r$ and *f* might have a simple relationship. Figure 1C and D show that they do. Under the rescaling $r \rightarrow r \cdot f$ all the data appear to merge, collapsing to a single curve that depends on the unifying factor $v:= r\cdot f$, or exploration velocity. This curve can be empirically characterized as

$$\tau (r\cdot f) = (r\cdot f)^a \cdot b$$

where *a* and *b* vary across cities and model types.

This relationship is robust to changes in model parameters. Figure S2 in the Supplementary Material shows that the relationship holds across initial infected proportions of $.1\%, 1\%, \text { and } 5\%$. Figure S3 shows that the same scaling collapse is achieved when the SI and SIR models are used, Figure S4 shows that it persists in higher values of $r\cdot f$ up to a certain limit, and Figure S5 shows that it persists for different values of the disease parameter $R_0$, though the relationship appears stronger with higher (as opposed to lower) $R_0$ values. These findings suggest the $r \rightarrow r\cdot f$ collapse persists across multiple disease processes.

**Figure 2:**
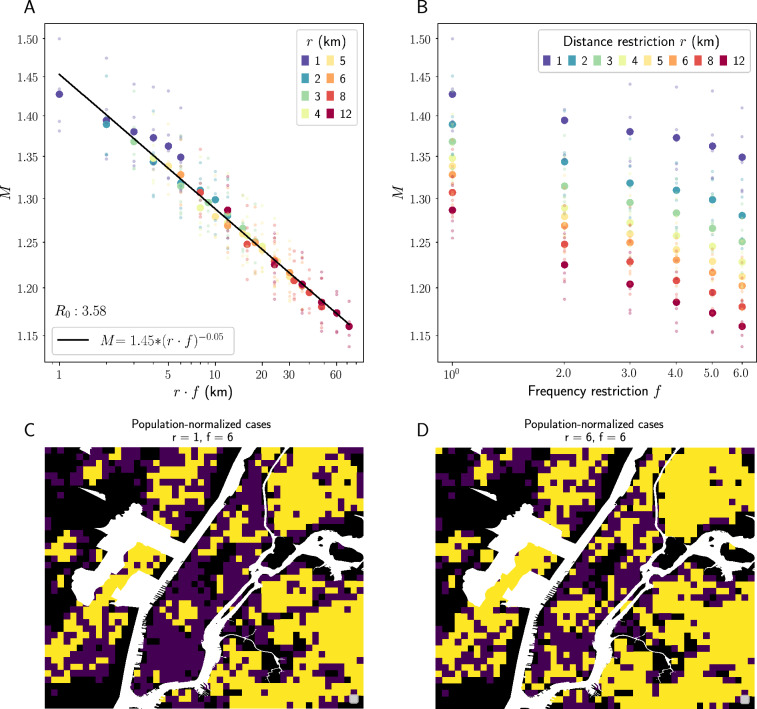
Scaling collapse of spatial dispersion of infections in NYC SEIR simulations. Top row: spatial dispersion *M* plotted against the product $r\cdot f$ (left) and plotted against maximum frequency restriction *f* for different values of *r* (right). Here, *M* is calculated using a radius of $k = 700$ meters. Both plots have logarithmic scales on both the x and y axes. We see a similar scaling collapse to that which we saw in $\tau$. $R^2$ of the best-fit line in Panel (**A**) is .982. Bottom row: this relationship is visually represented in a map of our NYC study area divided into approximately 400 m grid cells, where yellow grid cells have at least one infected agent living inside them and purple grid cells have no infected agents. We see geographic spread of infection for two simulations with the same *f* but different *r* values—infection disperses much more widely, especially through Manhattan, when *r* increases from 1 (left) to 6 (right) given fixed *f*.

After observing the relationship between $r\cdot f$ and characteristic time, we check whether there is a relationship between $r\cdot f$ and spatial dispersion of the disease. We analyze the spatial concentration of infected persons in our New York City simulations using the *M* function developed by Marcon and Puech, which is calculated in relation to some radius *k* around each agent ^17^—see Methods for a detailed description of this statistic. High *M* indicates tighter clusters of cases; low *M* indicates a more homogeneous distribution of cases across space. We find that for a given radius *k*, as *r* and *f* increase, *M*(*k*, *r*, *f*) decreases: infections become consistently more dispersed across the city (see Fig. 2A for this relationship when $k=700$m). As with epidemic size, under the rescaling $r \rightarrow r \cdot f$, this relationship collapses to a single curve, as shown in Fig. 2B. This indicates that restricting $r\cdot f$ is more effective than restricting *r* alone at containing geographic spread of disease, just as with slowing epidemic spread. This relationship is robust to the choice of *k*. As *k* increases, *M*(*k*, *r*, *f*) decreases (as would be expected—the larger radius you look at around an agent, the more representative sample of the whole population you will capture) but stays significant and maintains its relationship to $r\cdot f$—see Figure S6 in the Supplementary Material. This relationship is demonstrated visually in Fig. 2C and D, which show geographic spread of infection for two simulations which have the same *f* but different *r* values: in Panel C, where $r = 1, f = 6$, spread is more tightly clustered, especially in Manhattan, than in Panel D, where $r = 6, f = 6$ and the infection has dispersed more widely throughout the study area.

### Mechanisms

In order to illustrate the potential mechanism by which $r\cdot f$ determines disease spread time, we assess the relationship between $r\cdot f$ and both number and variance of contacts between agents in our simulations. Think of our simulations as a network, where agents are nodes and edges are formed whenever two agents come within some distance $\epsilon$ of one another, enabling disease transmission. It is a known result of the SIR model that speed at which a disease spreads through a network of individuals depends not only on the average degree, or number of contacts per individual $\langle k\rangle$, but also on the variance of contacts (via $\langle k^2\rangle$, or mean squared degree) according to the following relation:^18^

1 $$\begin{aligned} \tau = \frac{\langle k\rangle }{\langle k^2\rangle - (\gamma + \beta )\langle k\rangle }, \end{aligned}$$

where ${\tau }$ is the characteristic time, or time it takes for the disease to reach $1/e = 36\%$ of the population, $\gamma$ is the daily recovery parameter and $\beta$ is the infection parameter. This relation isn’t perfectly applicable to our context—it assumes exposure to all contacts at all timesteps, whereas our simulations incorporate movement in space and thus non-constant degree $\langle k\rangle$ over time. However, it is valuable in illustrating the effects of restricting *r* and *f* in our dataset. Restricting radius of travel *r* and frequency of return *f* affects both $\langle k\rangle$ and $\langle k^2\rangle$. The closer you stay to home and the fewer trips you take, the fewer unique individuals you have the opportunity to encounter (reducing average number of contacts $\langle k\rangle$). Further, as stricter restrictions are enforced, high-r, high-f trips are removed and the mobility patterns of high-frequency, long-distance travelers start to look more like the mobility patterns of low-frequency travelers who stay close to home (reducing variance of contacts $\langle k^2\rangle$).

**Figure 3:**
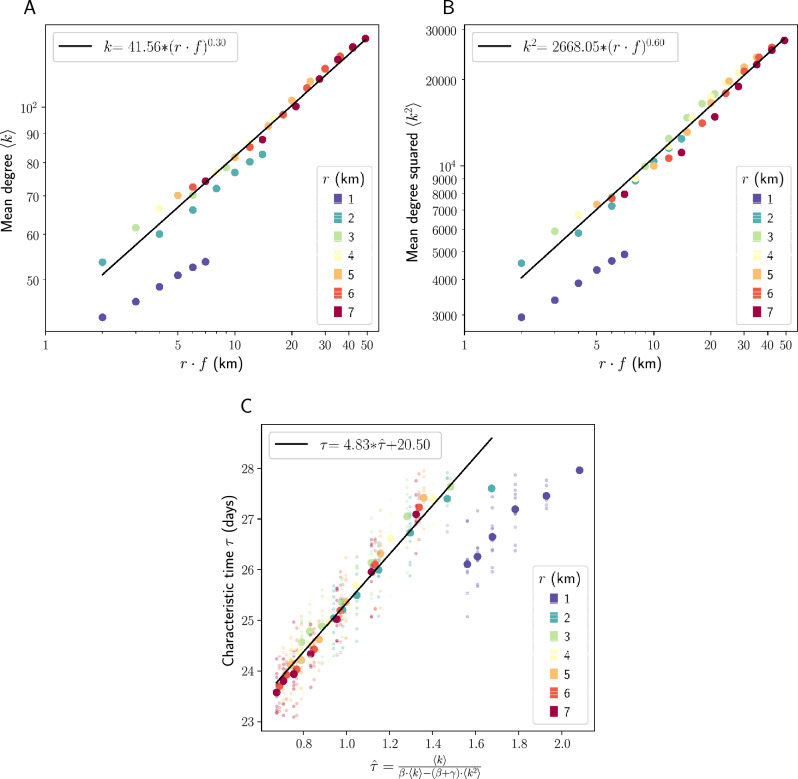
Relationship between spreading speed and number and variance of contacts. (**A**) $r\cdot f$ plotted against mean degree $\langle k \rangle$. (**B**) $r\cdot f$ plotted against mean degree squared $\langle k^2 \rangle$. Both (**A**) and (**B**) have logarithmic scales on both the x and y axes. (**C**) simulated $\tau$ plotted against $\hat{\tau}$ as predicted from Eq. 1 using degree and degree squared. Both mean number of contacts $\langle k \rangle$ and mean squared number of contacts $\langle k^2 \rangle$ of the agents in our dataset show a power law relationship with exploration velocity $r\cdot f$ when $r > 1$. The relationship between $r\cdot f$ and $\langle k \rangle$, $\langle k^2 \rangle$ can predict spreading speed $\tau$ using a simple relation that is a known result of the SIR model (Eq. 1). Best-fit lines are fit to data excluding $r=1$, which shows significant deviation from these relationships. $R^2$ values of best-fit lines are .977, .980, and .968 for (**A**), (**B**), and (**C**), respectively.

Figure 3 plots these relationships in our New York City data, showing exploration velocity $r\cdot f$ on the x-axis against average number of contacts $\langle k\rangle$ and average squared number of contacts $\langle k^2\rangle$ on the y-axis in Panels A and B, respectively. Again, we see a power law relationship, except at $r = 1$, where degree and degree squared grow more slowly than expected. Plugging both $\langle k\rangle$ and $\langle k^2\rangle$ into the relation above, we see that characteristic time as predicted by the degree distribution ($\hat{\tau }$) is proportional to the true characteristic time ($\tau$) that we see in our simulations—Fig. 3, Panel C. Taken together, these findings show that the shape of the relationship between $r\cdot f$ and $\tau$ can be predicted by decreases in number and variance of contacts between agents in combination with fundamental SIR modeling results.

**Figure 4:**
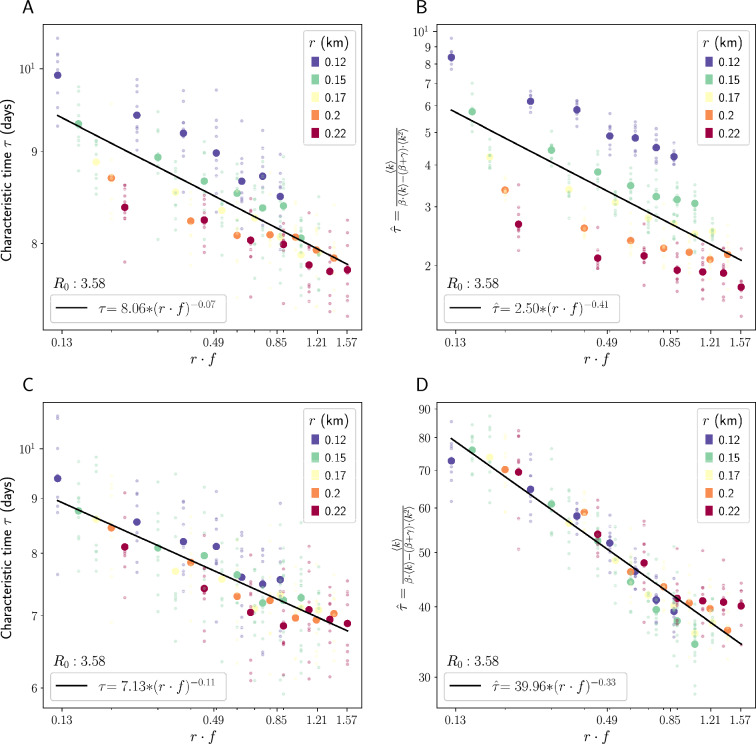
PEPR simulation results. (**A**) log $r\cdot f$ plotted against characteristic time $\tau$ from modified PEPR simulations with $P_{\text {travel}} = 1$. (**B**) log $r\cdot f$ plotted against predicted characteristic time $\hat{\tau }$ calculated using Eq. 1 and degree and degree squared from modified PEPR simulations with $P_{\text {travel}} = 1$. (**C**,**D**) analagous to Panels A and B but with $P_{\text {travel}} =.25$. All plots have logarithmic scales on both the x and y axes. When we run SEIR models across a set of trajectories $M_{\text {sim}}$ which have been created using the modified PEPR model with $P_{\text {travel}} = .25$, we see a similar relationship between $r\cdot f$ and $\tau$ to that in our real trajectories $M_{\text {real}}$. When we run SEIR models across trajectories which have been created using the original PEPR model (with $P_{\text {travel}} = 1$), we do not see this relationship. $R^2$ value of best fit lines are .690, .491, .875, and .924 for (**A**), (**B**), (**C**), and (**D**) respectively.

These results can be replicated by a simple modification of the preferential exploration and preferential return (PEPR) mobility model presented in previous literature^15^. Under the PEPR model as proposed in^15^, at each timestep, agents either explore a new location with a fixed probability $P_{\text {new}}$ or return to a previously-visited location with complementary probability $1-P_{\text {new}}$. In order to choose a new location, agents draw a radius of travel $\Delta r$ from a heavy-tailed distribution with exponent $P(\Delta r)\sim |\Delta r|^{-1-\alpha }$, with $\alpha = 0.55$ taken from^15^, and they draw an angle of travel $\theta$ with preference towards $\theta$ that are heavily visited by other agents (see Methods for details). It is demonstrated in^15^ that the PEPR model replicates the consistency of $r\cdot f$ across space that is seen in real mobility data. However, when we run our SEIR model on mobility trajectories simulated with the PEPR model, we do not see the same relationship between $r\cdot f$ and $\tau$ that we see in our real data (see Fig. 4C and D). This is because, in the original PEPR model, agents are always moving from place to place without necessarily returning home, whereas in our real mobility trajectories, time spent close to home (as enforced by our mobility restrictions) means less exposure to new, unique contacts and more exposure to the same neighbors. This leads to the PEPR model being unable to replicate the changes in $\langle k \rangle$ that are associated with loosening or tightening radius restrictions, as seen in Fig. 4A and B. In order to correct for this, we add a probability of travel $P_{\text {travel}}$—at each timestep, with probability $P_{\text {travel}}$, the agent travels to a new location according to the protocol above, whereas with complementary probability $1-P_{\text {travel}}$ the agent stays or returns home.

Figures 4C and D show the results of rerunning the same SEIR simulations that were run on our real mobility traces $\mathscr{M}_{\text {real}}$ on a set of simulated trajectories $\mathscr{M}_{\text {sim}}$ derived from the modified PEPR model with $P_{travel}$ set to .25, the true probability of travel in our real trajectories $\mathscr{M}_{\text {real}}$ (see Methods for details). We see the same power law relationship between $r\cdot f$ and both $\tau$ and $\hat{\tau }$ that we see in our simulations over $\mathscr{M}_{\text {real}}$, although the simulated results are less precise than the results we see in $\mathscr{M}_{\text {real}}$. We also replicate the simulations with $P_{\text {travel}} =.40$ and see similar results (see Figure S7 in the SM). This indicates that the modified PEPR model is able to replicate the relationship between exploration velocity and speed of epidemic spread.

## Discussion

The significance of $r\cdot f$ was revealed in recent work^15^ on human mobility patterns: the number of people who visit a given location *f* times from a distance *r* during a certain reference period follow an inverse square law $N(r,f) \propto 1/(rf)^2$. We suspect the $\tau (r\cdot f)$ relationship found in this paper derives from this inverse square law. We have explored the relationship between exploration velocity and network contacts, but deriving the exact mechanism linking the inverse square law to $\tau (rf)$ is an open question for future work.

An implication of the $\tau (r\cdot f)$ relationship is that restricting short, high frequency trips in a simulated environment does as much to slow disease spread as restricting long, low frequency trips. Agents in our simulations that take multiple trips around their neighbourhood spread disease as quickly as a those taking a single, long trip to the city center. This is interesting academically, but more importantly, it has potential implications for policy: it means that restricting travel distance *r* but ignoring travel frequency *f* could be ineffective in containing the spreading of a disease.

That said, an important limitation of this study is that it restricts exploration velocity in a very specific way, removing trips that extend beyond a certain *r* or *f*. In reality, it is unclear how people would respond to policies that aim to restrict exploration velocity. True behavioral responses to different travel policies and what policies or mechanisms would be required to change individuals’ behavior in a way that reduces exploration velocity are areas for future research. Further limitations of this study include the inherent limitations of simple epidemiological modeling—it is known that the SI, SIR, SEIR and other classic disease models make assumptions which bound their accuracy^19–21^—and of cell phone-derived data, which may under-represent certain socioeconomic groups^22^ and is likely to exclude children. The estimates for the model parameters likely carry error^23^, and we also assumed that each agent was identical and that each exposure within a set radius carried equal likelihood of infection.

It would be valuable in future work to explore whether these results hold under more complex disease transmission models or with different mobility datasets that may cover populations who are underrepresented in ours. Replicating this study with a mobility dataset that covers a longer timeframe would also be valuable, for two primary reasons. First, due to the limited timeframe of our dataset, we are only able to study pairs of initial and final thresholds that can be reasonably reached within 28 days and we are only able to use *r* and *f* restrictions for which a substantial number of users exceed the restriction (or the restriction would have no impact on our mobility traces)—see Figure S4 in the SM. Relatedly, by nature of our dataset, our simulations are limited to 28 days in length. This does not allow for a wide range of characteristic times, which makes it more difficult to establish a robust power law relationship. A replication study with a longer timeframe would allow the limits of the observed relationship to be more robustly tested.

Moreover, the scale of our analysis is restricted to the city level (because our datasets are collected at this level) and so our findings do not necessarily generalize to the perhaps more policy-relevant case of country-level and international travel. If the $r\cdot f$ collapse, or some form of inverse relation between *r* and *f*, holds for international mobility patterns, then, as at the city scale, lax distance limits could be compensated by strict frequency restrictions. This is a bold hypothesis, which should be tested in future work. Metapopulation disease models^24–26^, with their convenient trade off between realism and parsimony, seem like a good theoretical starting point for this effort.

Despite these limitations, we believe our results reveal novel insights and bring up an important component of human mobility that could prove useful for future modelling of COVID-19 and other epidemic diseases, as well as for policy design. In the current COVID-19 pandemic and in future pandemics, evidence-based policies at the city scale (not to mention the national or international scale) are needed to mitigate speed and intensity of disease spread. Our results have the potential to help this effort by opening up investigation into the relationship between exploration velocity and epidemic spread. They indicate that the exploration velocity of a city’s inhabitants $r\cdot f$ must be bounded—to bound distance *r* but not visitation frequency *f* leaves potential for disease containment unmet. Furthermore, and more optimistically, if a bound on exploration velocity is possible and effective, it would mean that strict distance restrictions at the city or town level—as adopted by, e.g., Ireland and Italy at the beginning of the infection—are perhaps unnecessary^6^. Given a desired bound on epidemic speed $\tau _{bound}$, a large *r* could be offset by a small *f*; allowing citizens to travel infrequently to distant services (doctors, hospitals etc) may be safe. This inverse relation between travel distance and frequency ($r \propto \tau _{bound} / f$) could also vitally inform remote working policies supporting the hypothesis that working from home multiple days per week— and thereby limiting the visitation frequency to workplaces—helps prevent the spread of disease.

## Methods

### New York City data

Individuals’ movements in New York City are inferred from GPS traces collected from mobile phones by the company X-Mode over a span of one month (February 2020). The raw data contains about 479,163 anonymized users; our analysis uses 10,000 users randomly selected from those that appear in the dataset every day in the month of February.

### Dakar data

The Dakar dataset is based on anonymized Call Detailed Records (CDR) provided by the Data for Development (D4D) Challenge. The detailed information of this dataset is provided in^27^. Here, we use SET2, which includes individual trajectories for 300,000 sampled users in Senegal, and after the preprocess, we have 173,000 users and 173 cells in Dakar region during two weeks of January, 2013. We subselect for users who appear at least 200 times in the dataset to ensure that we have adequate information about their trajectories over the two weeks.

### Data preprocessing

The X-Mode data from NYC is generated on an very fine spatial and temporal scale, with exact latitude and longitude coordinate updates as frequently as every second. The CDR data from Dakar, on the other hand, are generated only for voice calls, text messages or data exchanges and therefore have limited resolution in time. The geographic location of the cell towers and their density determines the accuracy of location measurements through triangularization techniques. Therefore, the trajectories extracted from CDRs constitute a discrete approximation of the moving population *M*(*x*; *y*; *t*). There are several steps in preprocessing of the data before it can be suitable for use in our analysis, which vary between the X-Mode data and the CDR data.

The main steps for the NYC data are: (i) We use density-based spatial clustering of applications with noise (DBSCAN) to group tightly-clustered latitude/longitude pairs in each individual’s trajectory into locations^28^. If a cluster of at least five latitude/longitude points exists such that no point is more than .0004 degrees (about 56 m) from two other points in the cluster, those points are grouped together as a single location. (ii) Each agent is assigned the DBSCAN cluster it visits most as its home location. (iii) We drop all locations in the trajectory that have been visited for less than a minimum time $\tau _{min} = 15{\text { min}}$. (iv) In order to restrict travel distance *r*, we calculate distance between locations by the haversine formula, which derives the great-circle distance between two points on a sphere. All locations that are more than *r* km from an agents home location are removed from their trajectory. (v) In order to restrict travel frequency *f*, for each DBSCAN cluster that an agent visits more than *f* distinct times (where distinct visits are determined by an agent leaving a location and then coming back to it), we randomly select *f* visits to include in their trajectory and drop the rest.

The main steps for the Dakar data are: (i) We view each cell tower as a different location in the city. (ii) For each person, we determine the home location as the cell tower location which has been visited for the most cumulative time. By summing over all days in a given time window, one can find the home cell with high level of confidence for the majority of subjects. (iii) We drop all locations in the trajectory that have been visited for less than a minimum time $\tau _{min} = 10{\text{ min}}$. iv) In order to restrict travel distance *r*, we calculate distance between cell towers by the haversine formula, which derives the great-circle distance between two points on a sphere. All cell towers that are more than *r* km from an agent’s home location are removed from their trajectory. (v) In order to restrict travel frequency *f*, for each location that an agent visits more than *f* distinct times (where distinct visits are determined by an agent leaving a location and then coming back to it), we randomly select *f* visits to include in their trajectory and drop the rest (excepting visits to an agent’s home location, which are not restricted).

The duration of stay, frequency, and distance criteria on defining cell visits yields a list of cells visited by that subject over the study period for a given frequency restriction *f* and distance restriction *r*.

### Simulation details

We run an agent-based SEIR, SIR, and SI models with $N = 10,000$ agents. In order to study the characteristic time of disease spread, we set our initial infected proportion, $i_0$, to a high enough value that the disease will reach 36% of all susceptible individuals within our study time frame (which is naturally restricted to 14 days within our Dakar data and 28 days within our NYC data). Empirically, we find that the minimum value is $i_{0\text {min}}$ = 10% for New York City and for Dakar in our SEIR models and 1% in SI and SIR models—see Figure S2 in the SM for an analogous exercise that lets us explore lower initial infected populations. Each agent is assigned the trajectory of a real person from our dataset, with location updated every 900 seconds (15 min) for the NYC simulations or every 600 seconds (10 min) for the Dakar simulations. At each time step, each user’s location is updated according to their assigned trajectory and infection status is updated according to the following parameters, drawn from Chen 2020^16^’s estimates of R0 = 3.58, incubation period = 5.2 days, and infection period = 5.8 days:

- $\beta =$ daily transmission parameter $= \frac{3.58}{5.8} = .617$
- $\sigma =$ daily rate at which an exposed person becomes infective $= 1/5.2$
- $\gamma =$ daily recovery parameter $= 1/5.8$

Let *s* be the number of time steps in a day. We then transform the above daily parameters into timestep parameters as follows:

- $\beta ^* =$ time step transmission probability $= \beta /s$
- $\gamma ^* =$ time step recovery probability $= 1 - \root s \of {1-\gamma }$
- $\sigma ^* =$ time step probability that an exposed person becomes infective $= 1 - \root s \of {1-\sigma }$

In addition, we reproduce our results with disease parameters estimated for the more highly-contagious Delta variant of COVID-19 ($\beta = 1.42$, all other parameters remain the same,^29^) and the 2009 H1N1 influenza strain ($\beta = .913, \gamma = 1.6, \sigma = 1$,^30,31^).

Finally, let $I_{\text {local}}$ and $N_{\text {local}}$ be the number of infected agents and total agents within a 190 meter radius of the agent’s current location for the NYC data or within the same cell tower location for the Dakar data. Then, transition probabilities are:

- $$\mathbb {P}[S\rightarrow E] = \beta ^* * \frac{I_{\text {local}}}{N_{\text {local}}}$$
- $$\mathbb {P}[E\rightarrow I] = \sigma ^*$$
- $$\mathbb {P}[I\rightarrow R] = \gamma ^*$$

In the SIR and SI models, if an agent becomes infected on a given day, they will become contagious at the start of the next day.

### Quantifying dispersion

We use the M function developed in^17^ to quantify spatial dispersion of disease in our New York City simulations for a given *r*, *f*. The M function is calculated as follows: for each infected agent *I* and some radius *k*, we calculate the ratio between the proportion of agents within *k* of *I* which are infected to the proportion of agents in the total population which are infected. Summing this value over all infected agents *I* and dividing by $N-1$, where *N* is the number of infected agents, gives *M*(*k*), the M function evaluated at *k*. While M is generally analyzed as a function over all reasonable *k*, we evaluate the M function at a specific *k* in order to compare spatial dispersion across $r\cdot f$ values at that *k*, and then show that the relationship is robust to choice of *k*.

Confidence intervals for *M*(*k*) are obtained by Monte Carlo simulation—for a given epidemic size $\psi$, we randomly assign $\psi$ infections across the population 1,000 times and calculate $M_\psi$ each time. By taking the .025 and .975 quantiles of these simulated *M*, we form an upper and lower bound on $M_\psi$ under the null hypothesis that infections are randomly distributed across individuals. It is notable that our empirical *M* never reaches this confidence band, implying that spatial dispersion is significantly non-homogenous for every *k* and $\psi$.

### Robustness to model parameters

We demonstrate that the $\tau (r\cdot f)$ curve is robust to changes in the parameters $R_0$ by running our simulations with estimated transmission parameters for the 2009 H1N1 influenza strain ($\beta = .913, \gamma = 1/1.6, \sigma = 1$^30,31^) and the Delta variant of COVID-19 ($\beta = 1.42, \sigma = 1/5.2, \gamma = 1/5.8$,^29^). Figure S5 shows that, with these parameters, the $\tau (r\cdot f)$ relationship still holds.

### Preferential return model

The preferential return model proposed in^15^ is based off of that proposed in Song et al.^32^. We assume uniform residential population density: home locations are drawn uniformly from the area of study. At each time step, with some probability $P_{\text {new}}$, agents return to a location they have already visited; with probability $1-P_{\text {new}}$ they visit a new location with distance drawn from the empirical distance distribution and direction drawn uniformly at random. The probability $P_{\text {new}}$ is a function of the number of locations already visited:

$$P_{\text {new}} = \rho S^{-\gamma _{\text {new}}}$$

where *S* is the number of locations visited. The parameters $\rho$ and $\gamma _{\text {new}}$ are fit to the real data using least-squares regression (using the NYC dataset, we find $\rho = .500$ and $\gamma _{\text {new}} = .267$.)

In order to implement radius restrictions within the PEPR model we restrict the distribution from which $\Delta r$ is drawn and in order to implement frequency restrictions we delete all trips beyond frequency *f* to the same location. We set $P_{\text {travel}} = .25$, the average proportion of time spent away from home in $\mathscr{M}_{\text {real}}$, and run the PEPR simulations on a unit square for 500 timesteps.

We add the following modification to the model in^15^: while in the original PEPR model agents travel at each timestep, we set some probability $P_{\text {travel}}$ with which agents travel at any given timestep. Conversely, with probability $1-P_{\text {travel}}$, agents stay home.

### Human subjects considerations

All methods were performed in accordance with the relevant guidelines and regulations. This research does not fall under the definition of human subjects research, as data is anonymous and unidentified and was not collected for the purpose of this research. No one on the study team has access to subject identifiers—all processing of raw GPS trajectories, including home location estimation, is done within-simulation and neither saved nor seen by the researchers.

### Supplementary Information

Supplementary Figures.

## Data Availability

The data and code used in this study are available from the corresponding author (cate.heine@gmail.com) upon reasonable request and with permission of X-Mode Social, Inc.
