# Supplementary material for: Travel distance, frequency of return, and the spread of disease
Source: Sci Rep. 2023 Aug 28;13:14064. doi: 10.1038/s41598-023-38840-0 (PMC10462643; doi:10.1038/s41598-023-38840-0)
Supplement: Supplementary file 1 — Supplementary Figures. [file 41598_2023_38840_MOESM1_ESM.pdf]

Travel distance, frequency of return and the spread of disease

Cate Heine, Kevin P. O'Keeffe, Paolo Santi, Li Yan, and Carlo Ratti

Supplementary Material

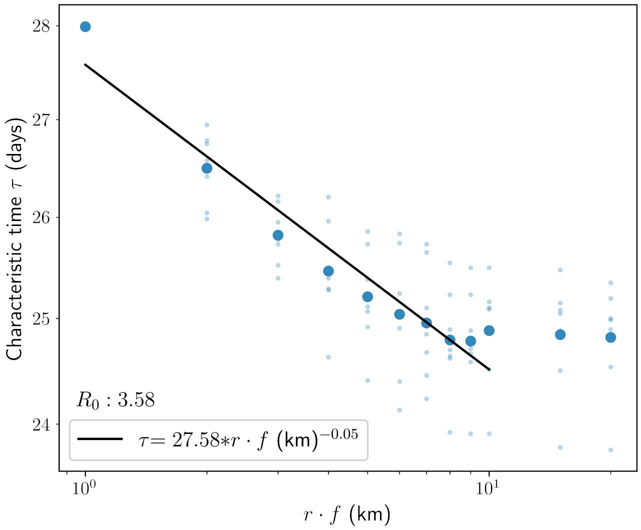

Figure S1. Relationship between $r \cdot f$ and τ when we limit the product $r \cdot f$ as opposed to r and f separately in SEIR, NYC simulation.. We run our simulations limiting the product $r \cdot f$—for each location r away from an agent's home, we only allow $r \cdot f / r$ visits. We see a similar relationship as in the primary formulation of our paper, up to a certain $r \cdot f$ value. Focusing on limiting r and f separately, as we do in the main body of the paper, allows us to understand if the driver of this relationship is r or f individually or the product itself. Understanding this limit at which the scaling relation no longer holds—and whether it is a fundamental aspect of the relationship between $r \cdot f$ and disease or an artifact of our data—is an important point for future exploration.

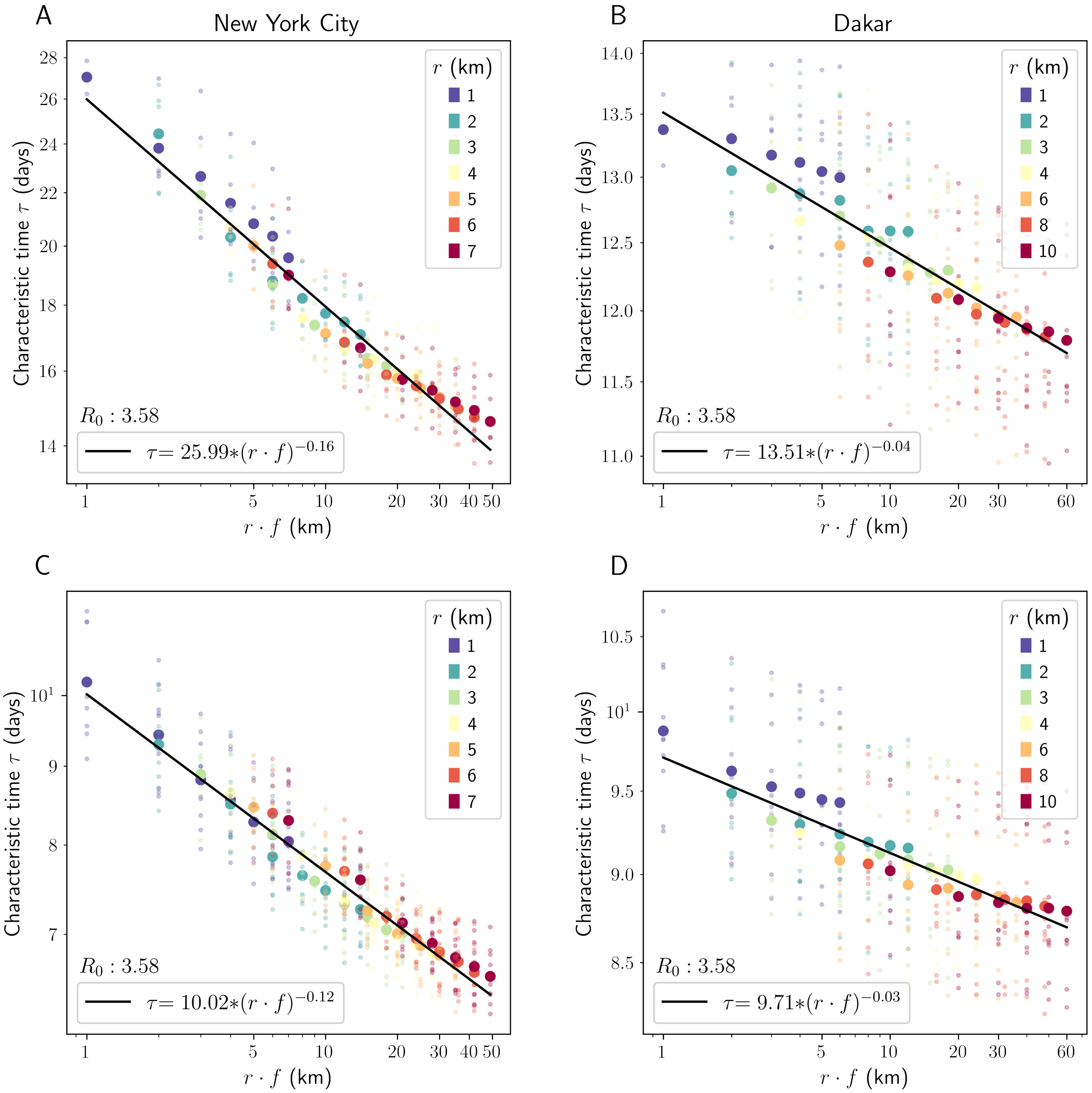

Figure S2. **Scaling collapse in SIR and SI model**. Top row: scaling collapse for SIR model with 1% initial infected populations. R^2 values for best-fit lines are, from left to right, .962 and .843. Best-fit line parameters are $a = -0.16$, $b = 25.99$ (NYC) and $a = -0.04$, $b = 13.51$ (Dakar). Bottom row, scaling collapse for SI model with 1% initial infected populations. R^2 values for best-fit lines are, from left to right, .976 and .835. Best-fit line parameters are $a = -0.12$, $b = 10.02$ (NYC) and $a = -0.03$, $b = 9.71$ (Dakar).

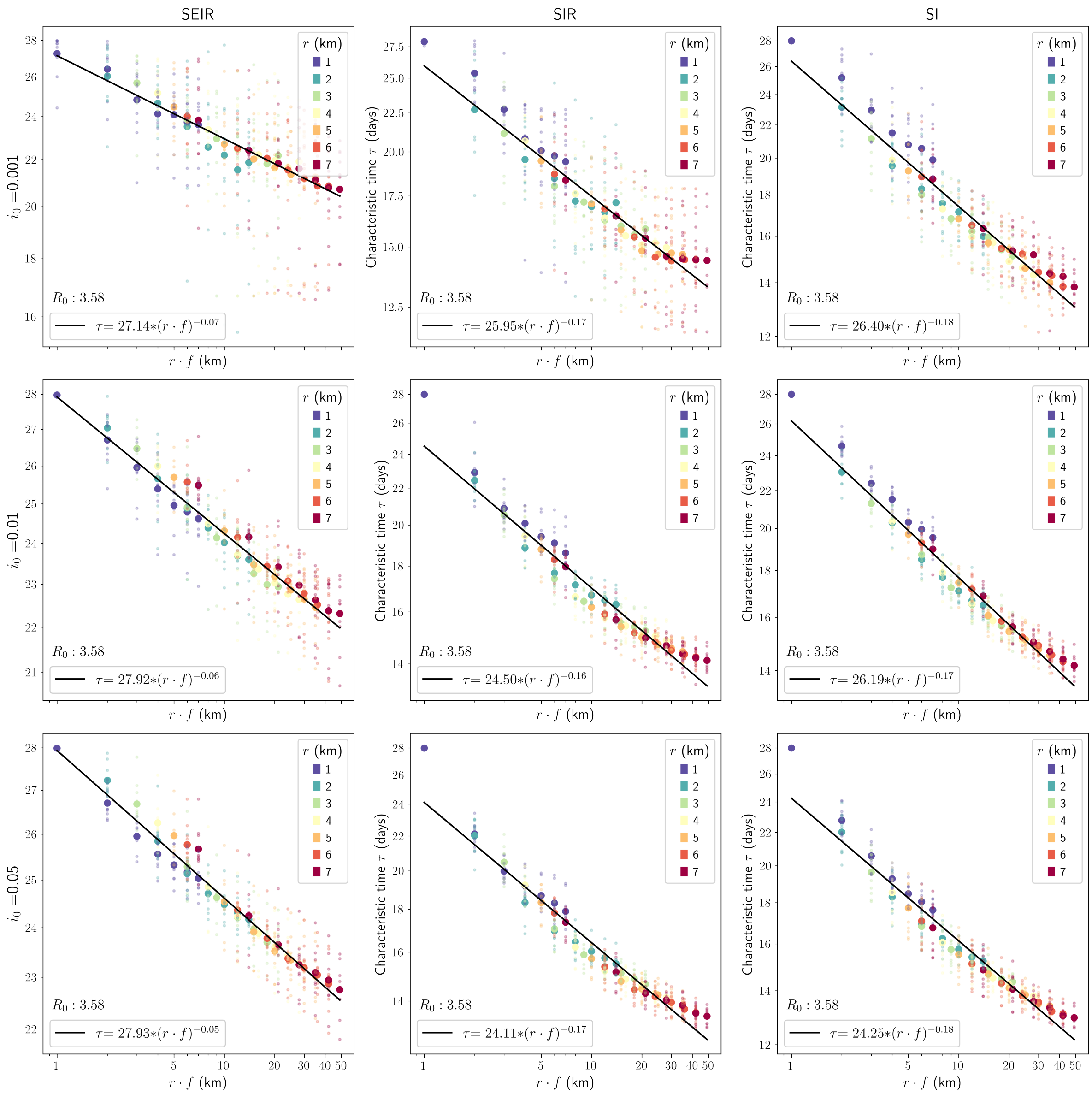

Figure S3. Persistence across i_0 values. Scaling collapse for NYC simulations with $i_0 = .1\%, 1\%, 5\%$ (rows 1, 2, and 3, respectively). In these simulations, total infected population never reaches $1/e \approx 35\%$ of the population, so we instead choose as our threshold the minimum peak infection size across trials to ensure that it will be reached by all simulations. The relationship between $r \cdot f$ and τ holds across all i_0 and model types, though variation is higher in lower i_0 simulations (see row 1 of the Figure).

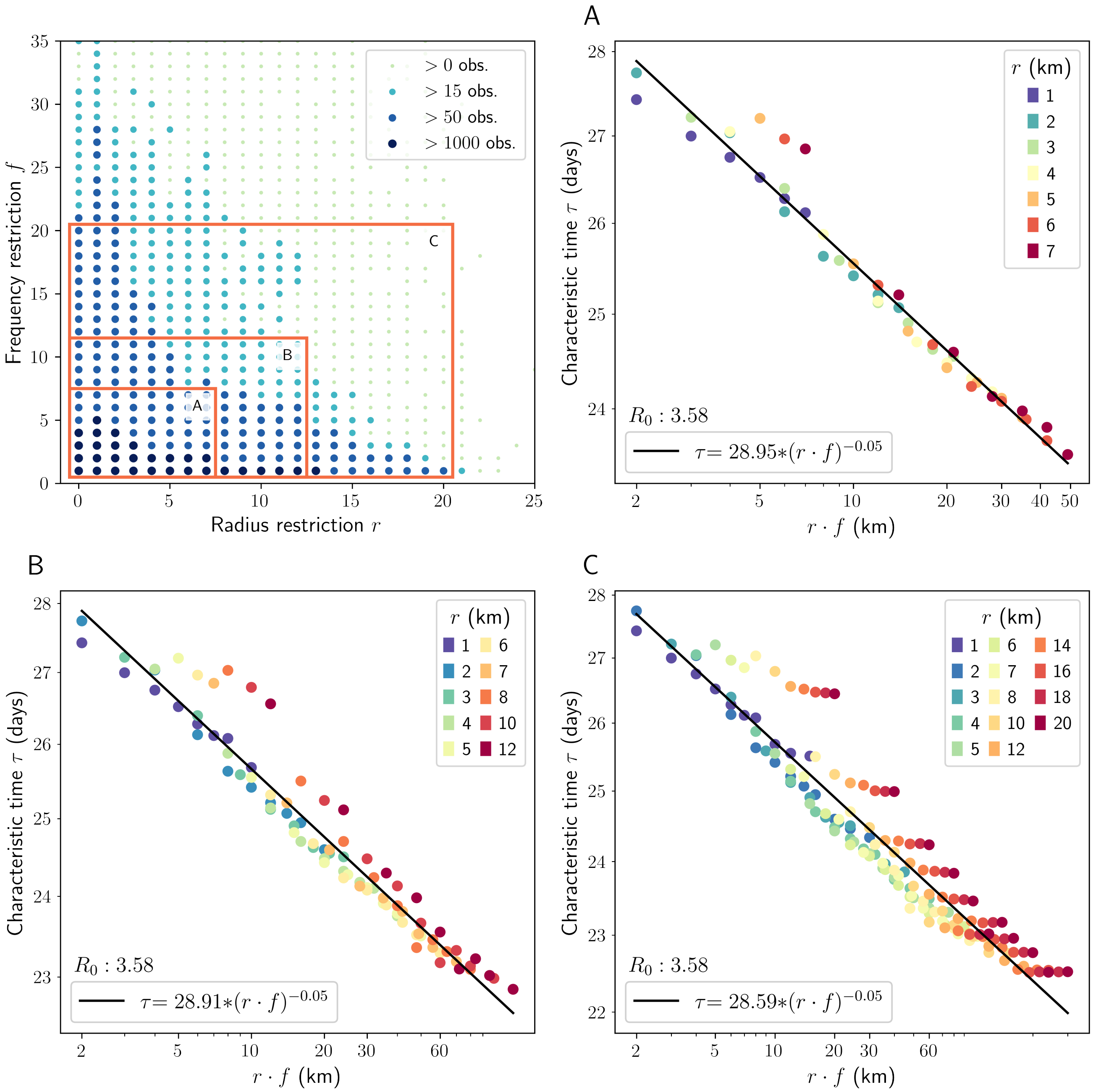

Figure S4. Relation limits in $r \cdot f$ for NYC data. In the main body of the paper, we show $r \cdot f$ collapse for $r, f <= 7$. These thresholds were chosen so as to ensure that we have a reasonable number of observations for each r, f pair. Top left shows, for each r and f pair, the number of individual-location observations within our dataset. For example, at $r = 5$, $f = 5$ there are 222 observations, each representing a given individual travelling to a given location that is 5 km away from their home 5 times. There are some r, f pairs that never show up in our data: for example, there are no observations in our dataset where an individual travelled to a given location 16 km away from their home 16 times. For $r, f <= 7$ (represented by box A on top left), all pairs of r and f are represented by at least 50 observations. If this threshold is lowered to 15 observations (which lets us expand our r, f values to to $r < 12, f < 11$, represented by box B on top left) then the scaling relation still holds (see plot B). However, if all $r, f < 20$ are considered, there are several high-r, high-f combinations which don't contain any observations at all and many that contain < 15 observations (see box C on top left). Thus limiting r and f at these high values has little to no effect on the dataset and the relationship with τ no longer holds (see plot C). This is a limitation of our dataset, given that our data only contains 28 days of data—if we observed the individuals in our dataset for a full year, there would certainly be more people visiting locations 16 km from their house 16 times. However, it is unclear to what extent it is also a limitation of the relation itself. Additional research using data collected over a longer period of time would be required to understand the true limits of the scaling relation in $r \cdot f$.

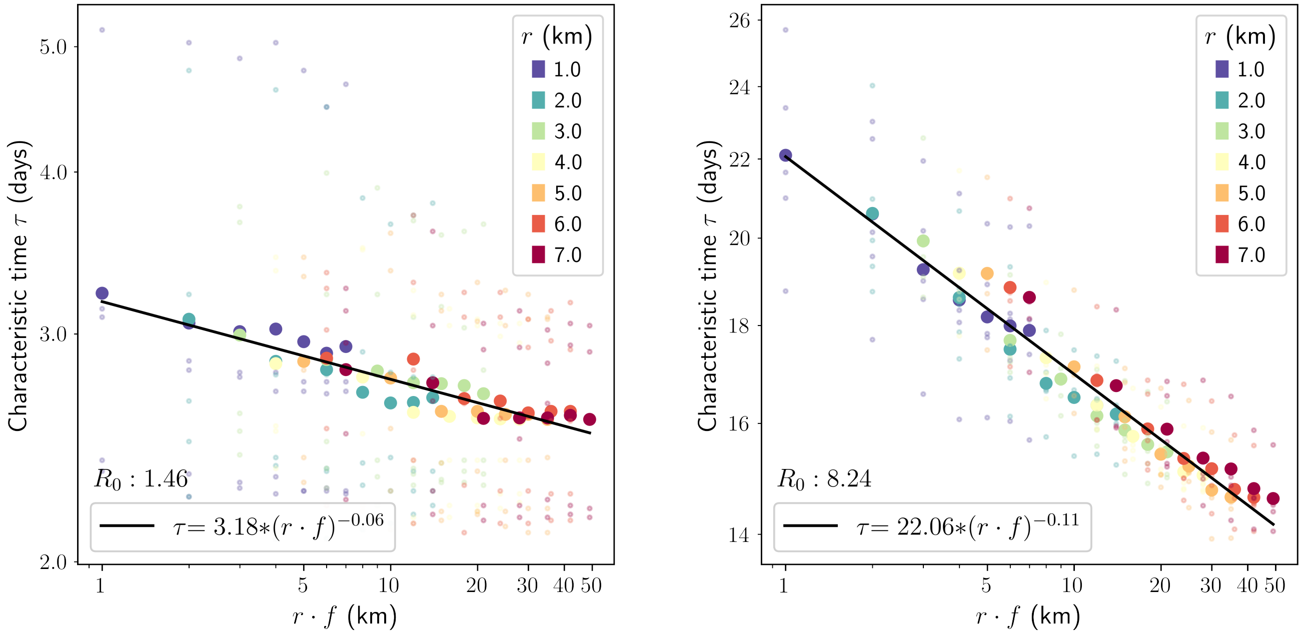

Figure S5. Characteristic times for SEIR simulations with lower $R_0 = 1.46$ (similar to the H1N1 epidemic) and higher $R_0 = 8.2$ (similar to the upper estimates of COVID-19 Delta variant contagion). R^2 of best-fit lines are .860 and .962, respectively. Simulations shown start with a 5% initial infected population. To calculate τ here, we used an infection threshold equal to the minimum peak infection size across r, f pairs in order to ensure that the threshold would be reached in the $R_0 = 1.46$ case. Note that, counterintuitively, characteristic time is lower in the $R_0 = 1.46$ case—this is because low-R_0 simulations have earlier peaks.

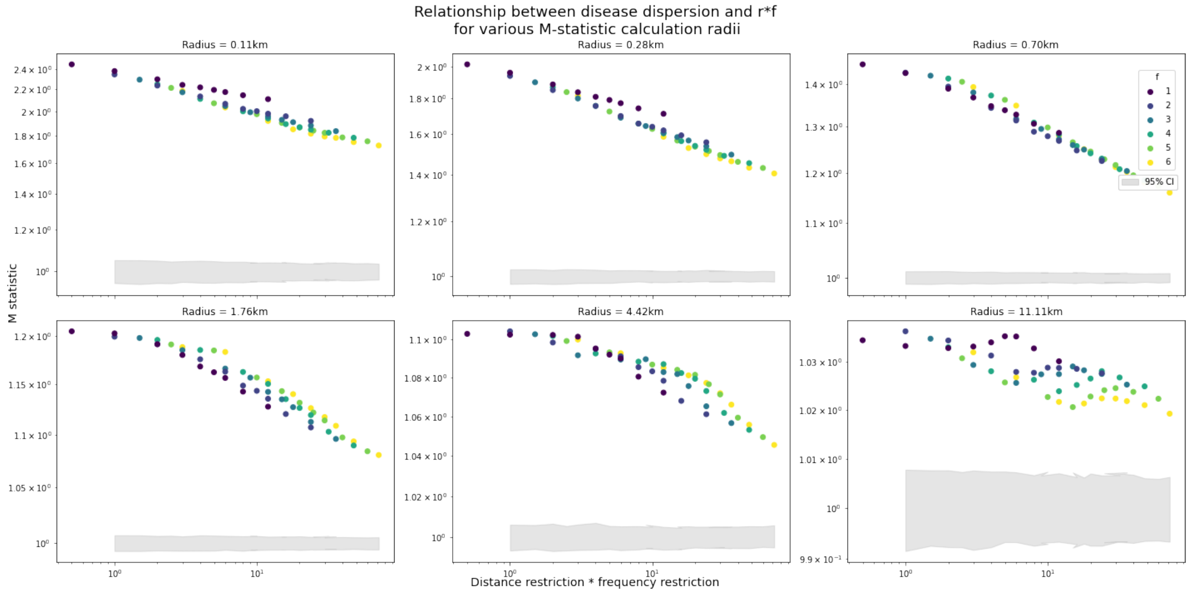

Figure S6. Collapse of spatial dispersion of infections for varius k in SEIR, NYC model. Spatial dispersion $M(k)$ shows a scaling relationship with $r \cdot f$ regardless of k. 95% confidence bands are shown in gray, indicating that the spatial clustering in infections remains significant across values of $r \cdot f$.

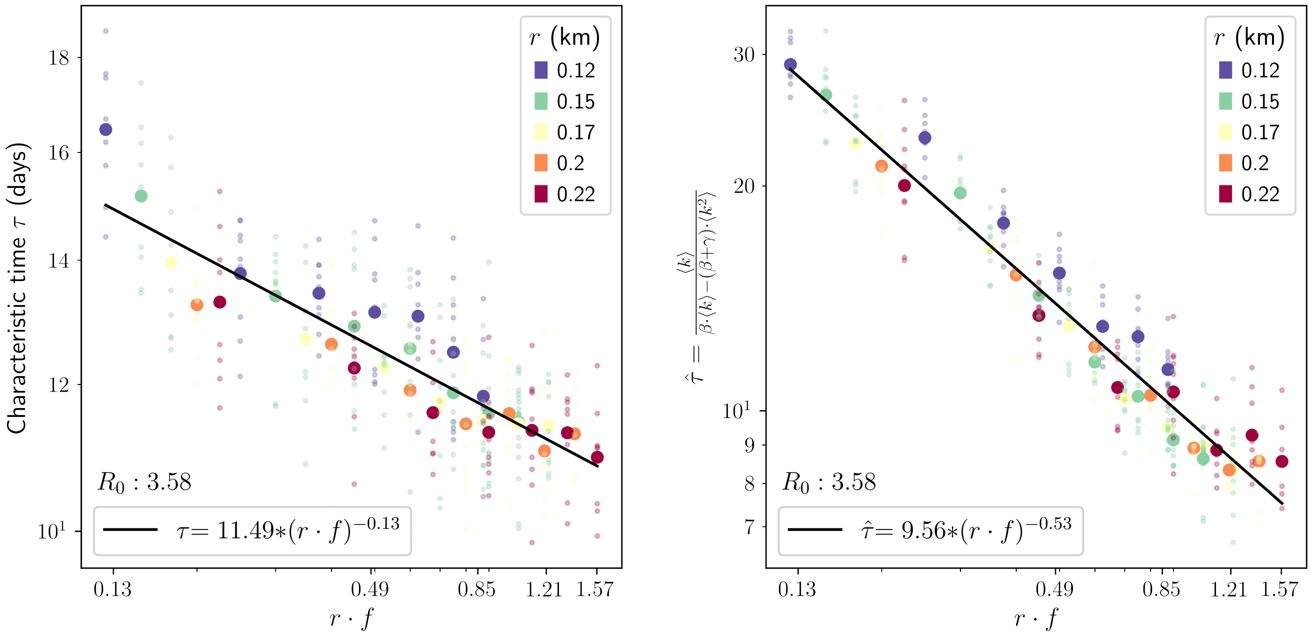

Figure S7. PEPR simulation results, where $P_{\text{travel}} = .40$. When we run SEIR models across a set of trajectories M_{sim} which have been created using the PEPR model with $P_{\text{travel}} = .40$, we see a similar relationship between $r \cdot f$ and τ to that in our real trajectories M_{real}. R^2 of best-fit lines are .869 and .955, respectively.
